# Heterogeneity among women with stroke: health, demographic and healthcare utilization differentials

**DOI:** 10.1186/s12905-021-01305-5

**Published:** 2021-04-17

**Authors:** Molly M. Jacobs, Charles Ellis

**Affiliations:** 1 Department of Health Services and Information Management, East Carolina University, 4340E Health Sciences Building, MS 668, Greenville, NC 27834 USA; 2 Department of Communication Sciences and Disorders, East Carolina University, Greenville, USA

## Abstract

**Background:**

Although age specific stroke rates are higher in men, women have a higher lifetime risk and are more likely to die from a stroke. Despite this increased severity, most studies focus on male/female differences in stroke onset, patterns of care and stroke-related outcomes. Given that stroke presents differently in men and women, mixed sex studies fail to fully capture heterogeneity among women with stroke and the subsequent impact on their outcomes. This study examined the sociodemographic characteristics, factors related to stroke incidence and post-stroke functional status between young (< 60) and old (≥ 60) women with stroke.

**Methods:**

Using 5 years of data from the National Health Interview Survey (NHIS), a nationally representative sample of US adults, cohorts of young and old women with stroke were identified. A set of demographic/lifestyle, health services utilization and health status characteristics were used evaluate within gender heterogeneity in three ways. First, disparities in population characteristics were assessed using Chi-Square and t tests. Second, young and old women with stroke were matched with women without stroke in their respective cohorts to determine differences in factors related to stroke incidence. Finally, the determinants of post-stroke functional limitation for the two cohorts were determined.

**Results:**

Young women with stroke were more likely to be Black, smoke regularly and frequently consume alcohol than older women. Young women were also less likely to engage with their health provider regularly or receive preventative health screenings. Diabetes, high blood pressure, high cholesterol and high BMI were correlated with an increased relative likelihood of stroke among older women. In contrast, family size, smoking frequency, alcohol consumption and sleep were correlated with an increased prevalence of stroke among young women. Although factors correlated with stroke varied between young and old women, health status and receipt of healthcare were the most significant determinants of post-stroke functional status for both cohorts.

**Conclusions:**

Health related characteristics were the primary correlates of stroke in older women, whereas post-stroke lifestyle and behaviors are the most significant correlates for younger stroke survivors. These findings suggest that while receipt of health services is essential for preventing stroke in both young and old women, providers should stress the importance of post-stoke lifestyle and behaviors to younger women at risk of stroke using approaches that may be different from older stroke women.

## Introduction

Stroke is the leading cause of long-term disability in the US with approximately 795,000 Americans newly diagnosed with a stroke annually [1]. There is a substantial literature indicating sex disparities in stroke and stroke-related outcomes [2]. Collective evidence indicates women are more likely to: (a) have a stroke over a lifetime due to longer lifespans [3, 4], (b) experience strokes at an older age [5], (c) have more severe strokes [6], (d) die of stroke [7], (e) have greater illness at the time of stroke [8] and (f) have greater long-term post-stroke disability [9] relative to men [10]. There are many reported reasons indicating why women are more likely to have a stroke including: women live longer than men, and older age increases stroke risk. Other important risk factors for stroke in women include hypertension, heart disease, diabetes mellitus [11–14], antiphospholipid antibodies [15], pregnancy, use of birth control containing estrogen [16] and migraine with aura [17–19]—conditions more common among women comparatively to men.

Since studies have also shown that women are typically 4 years older than men at the time of stroke onset, older age has been attributed to their reduced pre-stroke functional ability [20], greater overall chronic illness burden [21] and greater likelihood of more severe strokes [22–24]. Furthermore, because strokes typically occur later in life, other age-related processes are also occurring that result in greater biological aging in stroke survivors over and above their chronological age [25]. Consistently, age is cited as the primary determinant of outcome differentials. However, researchers have to date offered little evidence explaining differential outcomes among young stroke survivors, particularly women. This study supplements existing knowledge of women with stroke by examining differences in demographic/lifestyle characteristics, physical/mental health status and health services utilization between young (< 60) and old (≥ 60) women with stroke. This analysis provides a more accurate characterization of stroke in women than previous mixed gender studies by examining within sex heterogeneity. This framework explores differences female age cohorts to enhance understanding of the causal, characteristic and outcome disparities between young and old female stroke survivors.

Healthcare, demographic and health status characteristics used in this study were drawn from literature citing correlation with stroke. While the role of traditional risk factors in the pathogenesis of stroke in younger adults has been debated, several studies have demonstrated a high prevalence of traditional cardiovascular risk factors among young individuals with stroke, primarily high levels of alcohol consumption [26], smoking [11, 14, 18], physical inactivity [27], poor diet [28], and obesity [29, 30]. While recent studies have shown that ischemic stroke is increasing in younger adults, they have also demonstrated an increasing prevalence of those traditional stroke risk factors (hypertension, dyslipidemia, diabetes mellitus, tobacco use, and obesity) that were previously only common in older adults [31, 32]. It is believed that a higher prevalence of these traditional risk factors increases susceptibility to stroke from other causes in younger adults [33].

Little is known, about stroke among younger age cohorts and even less is known about stroke in young women. This study extends the current research regarding stroke in women by examining the differences in demographic/lifestyle, healthcare utilization, health status and post-stroke functional status among old and young women with stroke. A greater understanding of stroke in women and how various characteristics are related to the prevalence of stroke in women may help better identify high risk populations and target preventative strategies. Furthermore, analysis of differences among cohorts of women, rather than between men and women, may help clarify mechanisms of stroke in young and old women and potential differences in risk factors related to the incidence of early stroke.

## Materials and methods

### Database

Data from the 2014 through 2018 National Health Interview Survey (NHIS) was pooled to identify two cohorts of young (age less than 60) and old (age greater than 60) women. NHIS is a cross sectional household interview survey collected through geographically clustered sampling techniques. Information in the NHIS concerns the amount, distribution and effects of illness and disability in the United States and the services rendered for or because of such conditions in the noninstitutionalized civilian population. The main objective of the NHIS is to monitor the health of the US population through the collection and analysis of data on a broad range of health topics. The survey allows categorization of a variety of health characteristics by demographic and socioeconomic characteristics. Content in the NHIS is updated every 15–20 years, most recently in 2019 when it launched a redesigned content and structure that differs from its previous questionnaire design (1997–2018). Due to the redesigned 2019 sample, only data from 2014 through 2018 is included in this study due to comparability of survey items.

NHIS is a cross-sectional household interview survey targeting the civilian noninstitutionalized population residing within the 50 states and the District of Columbia at the time of the interview. The NHIS universe includes residents of households and noninstitutional group quarters. As the NHIS is conducted in a face-to-face interview format, a simple random sample of dwelling units would be too dispersed throughout the nation; as a result, the costs of interviewing a simple random sample of 35,000 dwelling units would be prohibitive. To achieve sampling efficiency and to keep survey operations manageable, cost-effective, and timely, the NHIS uses complex sampling techniques to select the sample of dwelling units for the NHIS. These methods partition the target universe into strata and clusters. Therefore, users of the public use NHIS files must utilize appropriately provided controls for clustering and stratification in addition to sampling weights to ensure that estimates are unbiased and standard errors are consistent. Additional information can be reviewed here: https://www.cdc.gov/nchs/nhis/methods.htm.

### Sample

This investigation utilized a historical cohort study design. The study cohort was constructed by pooling women from the 2014 through 2018 NHIS ≥ 18 years who had responded either yes or no to the question “Have you ever been told by a doctor or other health professional that you had a stroke?” (n = 65,092). While NHIS does not include information on respondent age at stroke diagnosis, it does provide their age at the time of survey response. Women were divided into two cohorts based on age—young, less than 60, and old, greater than or equal to 60. Strokes were assumed to have occurred prior to the time of interview.

Since the average of stroke in women is 72.9, the young cohort was characterized by below average age at stroke [34]. This study cannot rule out that women in the older cohort suffered a stroke prior to age 60. However, estimates suggest only five to six women, 0.4%, in the older category would be misclassified [17]. Among the 65,092 women who responded to the survey item on stroke diagnosis, only 4%, (2,379 women unweighted) reported having experienced a stroke—slightly below the national average. Over 70% were over age 60 (n = 1,715), with less than 30% below age 60 (n = 664).

### Variables of interest

Characteristics of interest were divided into four categories: (1) demographic/lifestyle, (2) health services utilization, (3) health status and (4) post-stroke functional status.

- *Demographic/Lifestyle characteristics* include race, region of residence, marital status, household size, smoking regularity, frequency of alcohol consumption and hours of sleep. These characteristics, as referenced above, are correlated with stroke and/or primary health outcomes.
- *Health services utilization* is characterized by receipt of health services within the last 12 months including blood pressure check, cholesterol screening, prescribed medications, total number of office visits, total number emergency room visits, total number of surgeries, having a regular place to go when sick and the most recent time respondents saw or talked to a health professional. These services were received post-stroke and NHIS does not provide an indicator capturing pre-stroke receipt of care. However, health services literature suggests that individuals establish patterns of health services utilization, particularly preventative care, relatively early in life [35–38]. As long as individuals maintain some form of health insurance (all women in the panel were insured), their patterns remain relatively consistent over the life course [36, 37]. Therefore, this study contends that previous receipt of health services in highly correlated with present receipt of health services apart from catastrophic accident or event and the preventative care and screening patterns remain consistent [36].
- *Health status* describes both physical and emotional well-being. Indicators for diagnosis of diabetes, hypertension and high cholesterol and the frequency of feeling worthless, hopeless or depressed. These conditions were reported at the time of the survey rather than the time of stroke. However, given their chronic nature and duration of development, they likely existed for many years and developed prior to the stroke event.
- *Post-stroke functional status* is described by presence of a functional limitation, suffering from a chronic condition or feeling as though stroke-related problems cause difficulty with activities. These items are derived directly from survey items regarding functional status.

### Statistical analysis

All three phases of analysis were performed using the SAS statistical package (SAS System for Windows, Version 9.4, Cary NC, USA). First, Pearson Chi-Square Statistics and independent sample t-tests investigated disparities in demographic/lifestyle characteristics, healthcare utilization, health status and post-stroke functional status between young and old women with stroke. Second, the correlation between healthcare utilization, demographic/lifestyle traits and health status and prevalence of stroke was calculated for the young and old cohorts of women using Cox proportional hazards regression on a match cohort of young and old women with and without stroke. Matching was performed using propensity scores derived from age, race, region of residence, education, household size, and marital status with replacement. Finally, the impact of health status, healthcare utilization and demographics/lifestyle on post-stroke functional status was assessed with multiple logistic regression for young and old stroke sufferers.

## Results

Mean prevalence values for all demographic, health status, healthcare utilization and functional status characteristics are listed in Table 1 for the young and old cohorts. Comparative sample statistics indicating significant differences between the cohorts are provided in Table 1.

**Table 1:** Descriptive statistics for old and young women with stroke. *Source*: 2014–2018 National Health Interview Survey

	Old (Age ≥ 60)		Young (Age < 60)		Test of significant difference between cohorts	
	N = 1715		N = 664			
	Mean	SD	Mean	SD	Chi-square/T statistic	P value
*Demographic/lifestyle characteristics*						
Age	74.93	7.76	49.06	9.12		
Black	0.17	0.37	0.24	0.43	16.77	< .0001
South	0.42	0.49	0.46	0.50	4.14	0.0373
Married	0.25	0.43	0.33	0.47	13.62	0.0001
Family size	1.67	1.05	2.26	1.39	− 11.18	< .0001
Smoker	0.46	0.50	0.53	0.50	9.33	0.0019
Days of alcohol	4.28	27.37	3.32	17.52	0.73	0.463
Sleep	7.50	2.04	6.72	2.16	8.09	< .0001
*Health services utilization*						
BP check	0.96	0.19	0.91	0.28	28.38	< .0001
Chol check	0.93	0.26	0.81	0.39	66.2	< .0001
RX	0.95	0.21	0.89	0.31	31.94	< .0001
Office visits	4.06	2.33	4.29	2.69	− 2.05	0.0407
ER visits	0.77	1.12	1.13	1.49	− 6.38	< .0001
Surgeries	1.77	0.45	1.74	0.47	1.44	0.1501
Speak to provider	0.92	0.28	0.84	0.36	27.1	< .0001
Regular care facility	0.98	0.13	0.98	0.13	0.01	0.9146
*Health status*						
Hypertension	0.92	0.28	0.91	0.28	0.11	0.7216
Diabetes	0.29	0.45	0.26	0.44	2.89	0.0999
Cholesterol	0.66	0.47	0.47	0.50	70.06	< .0001
BMI	28.15	7.03	30.97	8.97	− 7.93	< .0001
Hopeless	0.05	0.23	0.11	0.32	24.52	< .0001
Worthless	0.06	0.23	0.09	0.29	12.04	0.0002
sad	0.07	0.25	0.13	0.34	23.04	< .0001
*Quality of life*						
Stroke problems	0.27	0.44	0.31	0.46	3.72	0.0493
Functional limitation	0.89	0.32	0.78	0.41	42.27	< .0001
Chronic conditions	0.99	0.08	1.00	0.06	0.51	0.4804

### Cross-cohort comparisons of key outcome variables

#### Demographic/lifestyle

Young women with stroke are 24% Black, compared to only 17% in the older cohort—a statistically significant difference (χ^2^ = 16.77, p < 0.001). They are also more likely to live in the South (χ^2^ = 4.14, p = 0.0373), be married (χ^2^ = 13.62, p = 0.001) and smoke cigarettes (χ^2^ = 9.53, p = 0.0019). They also live in larger houses, 2.26, than their older counterparts (1.67) (t-statistic = 11.18, p < 0.001) and sleep less per night (6.72, 7.50) (t statistic = 8.09, p < 0.001).

#### Healthcare utilization

The number of office visits, emergency department visits and surgeries in the past year was assessed between young and old women as well as routine screening for blood pressure, cholesterol and blood sugar. Surprisingly, older women visit the physician’s office (4.06 for older vs 4.29 for younger) and the emergency room (0.77 for older vs 1.13 for younger) less frequently than younger women with stroke (office visits, t statistic = 2.05, p = 0.0407; emergency room, t statistic = 6.38, p < 0.001). Older women have higher prevalence of blood pressure checks, cholesterol screenings, prescribed medication and speaking with their provider in the past 12 months.

#### Health status

While older women have a higher prevalence of physical health problems including of hypertension (0.92 vs 0.91) (χ^2^ = 0.11, p = 0.7216) diabetes (0.29 vs 0.26) (χ^2^ = 2.89, p = 0.0999) and high cholesterol (0.66 vs 0.47) (χ^2^ = 70.06, p < 0.001), younger women are more likely to have symptoms of poor mental health including feelings of hopelessness (0.05 vs 0.11) (χ^2^ = 24.52, p < 0.001), worthlessness (0.06 vs 0.09) (χ^2^ = 12.04, p = 0.002) and sadness (0.07 vs 0.13) (χ^2^ = 23.04, p < 0.001).

#### Post-stroke functional status

Younger women are more likely to report that stroke-related difficulties cause problems with activity (30% of younger vs 27% of older, t statistic = 3.72, p = 0.0493), while older women more frequently report overall functional limitations (78% for younger vs 89% for older, t statistic = 42.27, p < 0.001). However, there is no statistically significant difference in reporting of difficulties related to chronic conditions (t statistic = 0.51, p = 0.4804).

### Contribution of key outcome variables to stroke prevalence

To determine the relationship between demographics/lifestyle, health status and healthcare utilization on prevalence of stroke for old and young women, individuals with stroke in each cohort were matched with women without stroke based on age, race, region, and chronic conditions. Results from cohort specific models are provided in Table 2. Those estimates significantly correlated with stroke differ between you and old women.

**Table 2:** Impact of selected demographic, health care utilization and health status characteristics on the prevalence of stroke for young and old women. *Source*: 2014–1028 National Health Interview Survey

	Old cohort				Young cohort			
	Log likelihood	12,242.677			Log likelihood	4000.929		
	Estimate	Hazard ratio	95% CI		Estimate	Hazard ratio	95% CI	
Black	**0.43693**	1.548	1.27	1.887	0.23993	1.271	0.949	1.702
South	**0.36052**	1.434	1.231	1.671	**0.40848**	1.505	1.171	1.934
Married	0.16993	1.185	0.992	1.417	− **0.45589**	0.634	0.475	0.846
Family size	**0.16129**	1.175	1.088	1.269	**0.16642**	1.181	1.074	1.299
Smoker	**0.45495**	1.576	1.364	1.821	0.23637	1.267	0.982	1.634
Alcohol consumption	− 0.00124	0.999	0.996	1.001	− 0.00229	0.998	0.99	1.005
Sleep	− 0.01381	0.986	0.949	1.025	− **0.10492**	0.9	0.836	0.97
Prescriptions	**0.60867**	1.838	1.057	3.197	− 0.15134	0.86	0.492	1.503
Office visits	**0.03536**	1.036	1.001	1.073	**0.10224**	1.108	1.047	1.172
ER visits	**0.24169**	1.273	1.193	1.359	**0.23604**	1.266	1.179	1.36
Surgeries	− 0.1002	0.905	0.76	1.077	0.1019	1.107	0.815	1.505
Usual care facility	− 0.12029	0.887	0.439	1.791	− 0.29586	0.744	0.306	1.808
Hypertension	0.17422	1.19	0.923	1.535	0.27785	1.32	0.862	2.022
Diabetes	**0.20904**	1.232	1.046	1.453	0.18881	1.208	0.92	1.585
Cholesterol	**0.42376**	1.528	1.299	1.796	0.06734	1.07	0.83	1.379
BMI	**0.03254**	1.033	1.022	1.044	0.00263	1.003	0.988	1.018
Sad/depressed	**0.57341**	1.774	1.331	2.366	**0.45926**	1.583	1.115	2.248

Indicates significant at the 95% level in Cox proportional hazard model

Among older women, more frequent utilization of health services such as office visits (β = 0.04, p value = 0.0466, HR = 1.04), prescription medications (β = 0.61, p value = 0.0311, HR = 1.84) and emergency room (β = 0.24, p value < 0.001, HR = 1.27) visits was highly correlated with stroke compared to those with lower frequencies of utilization in their age cohort. Additionally, older women with confounding comorbid conditions like hypertension (β = 0.17, p value = 0.0179, HR = 1.19), diabetes (β = 0.21, p value = 0.0127, HR = 1.12), high cholesterol (β = 0.42, p value < 0.001, HR = 1.53) and higher BMI (β = 0.03, p value < 0.001, HR = 1.03) showed substantially larger relative prevalence of stroke.

Among younger women, frequent visits to physicians’ offices (β = 0.01, p value = 0.004, HR = 1.11) and the emergency room (β = 0.024, p value < 0.001, HR = 1.27) were significantly related to incidence of stroke. The largest correlates of stroke among younger women were household, demographic, and lifestyle characteristics. The magnitude of the hazard ratio was small compared to other characteristics. Women in the younger cohort living in the South (β = 0.41, p value = 0.0014, HR = 1.51) with larger families (β = 0.17, p value = 0.006, HR = 1.18) have higher prevalence. However, lifestyle characteristics appear to have the largest impact on stroke incidence. Smoking (β = 0.24, p value = 0.0486, HR = 1.27) and consuming alcohol (β = 0.00, p value = 0.0505, HR = 1.00) were positively correlated with incidence of stroke, while increasing health habits like sleep (β = − 0.10, p value = 0.0052, HR = 0.90) were negatively correlated with stroke. Interestingly, being married (β = − 0.46, p value = 0.002, HR = 0.63) was negatively correlated with stroke while feeling sad or depressed (β = 0.46, p value = 0.0103, HR = 1.58) was positively correlated with stroke incidence.

### Contribution of key variables to post-stroke functional status

To evaluate the determinants of post-stroke functionality among old and young women, three measures of functionality were assessed: overall functional limitations, stroke problems impeding activity and chronic conditions limiting behavior. Results are listed in Tables 3, 4 and 5. To observe associations with post-stroke functional limitations, regressions included a subset of characteristics to allow comparison of the determinants between cohorts. Interestingly, older and younger women experience similar determinants of post-stroke functionality. Interestingly, family size and being married is associated with a higher relative probability of having limited function or difficulty with activity. Indicating that individuals with limitations often live with other household members or that individuals in larger household rely on others to perform tasks that require mobility.

**Table 3:** Determinant of post-stroke functionality: stroke problem causes difficulty with activity. *Source*: 2014–1028 National Health Interview Survey

	Old cohort				Young cohort			
	Log likelihood		1037.05		Log likelihood		434.936	
	Estimate	Odds ratio	95% CI		Estimate	Odds ratio	95% CI	
Intercept	− 0.2525				**2.4929**			
Age	**0.0294**	1.03	1.01	1.05	− **0.0187**	0.981	0.956	1.008
Black^a^	− **0.5054**	0.603	0.401	0.907	0.0819	1.085	0.686	1.718
South^b^	0.1896	1.209	0.887	1.648	− 0.1188	0.888	0.572	1.379
Married^c^	0.1784	1.195	0.849	1.684	− 0.1172	0.889	0.524	1.51
Family Size	− **0.1647**	0.848	0.729	0.986	0.0527	1.054	0.851	1.306
Alcohol Consumption	− **0.00542**	0.995	0.99	0.999	0.026	1.026	0.989	1.065
Sleep	− **0.123**	0.884	0.825	0.948	− **0.1543**	0.857	0.776	0.947
BMI	**0.0142**	1.014	0.995	1.034	0.0128	1.013	0.993	1.034

Indicates significant at the 95% level in binary logistics regression

^a^Reference category: non-Black

^b^Reference category: West, Midwest, Northeast

^c^Reference category: Nonmarried

**Table 4:** Determinants of post-stroke functionality: overall functional limitations. *Source*: 2014–1028 National Health Interview Survey

	Old cohort				Young cohort			
	Log likelihood		758.755		Log likelihood		513.361	
	Estimate	Odds ratio	95% CI		Estimate	Odds ratio	95% CI	
Intercept	**2.7166**				**3.2203**			
Age	− **0.0372**	0.963	0.94	0.988	− **0.085**	0.919	0.896	0.941
Black^a^	0.3499	1.419	0.852	2.363	0.1059	1.112	0.583	2.119
South^b^	− 0.1241	0.883	0.589	1.325	− 0.3824	0.682	0.427	1.089
Married^c^	**0.5456**	1.726	1.095	2.718	**0.8046**	2.236	1.277	3.916
Family Size	− 0.1455	0.865	0.677	1.104	0.0507	1.052	0.878	1.26
Alcohol Consumption	− 0.00449	0.996	0.989	1.002	− 0.00112	0.999	0.991	1.007
Sleep	0.0275	1.028	0.959	1.101	**0.1219**	1.13	1.029	1.24
BMI	− **0.0788**	0.924	0.902	0.947	− **0.0519**	0.949	0.924	0.976

Indicates significant at the 95% level in binary logistics regression

^a^Reference category: non-Black

^b^Reference category: West, Midwest, Northeast

^c^Reference category: Nonmarried

**Table 5:** Determinants of post stroke functionality: chronic condition(s) cause limitations. *Source*: 2014–1028 National Health Interview Survey

	Old cohort				Young cohort			
	Log likelihood		51.505		Log likelihood		24.924	
	Estimate	Odds ratio	95% CI		Estimate	Odds ratio	95% CI	
Intercept	− **0.2245**				− 22.6241			
Age	− 0.00402	0.996	0.925	1.072	**0.2846**	1.329	1.03	1.715
Black^a^	0.4892	1.631	0.188	14.112	**2.2004**	9.029	5.378	15.158
South^b^	0.4239	1.528	0.323	7.224	− 0.1151	0.891	0.194	4.089
Married^c^	− 10.9313	< 0.001	< 0.001	< 0.001	**2.8394**	17.105	2.716	107.741
Family size	**0.2326**	1.262	0.844	1.888	− 1.2798	0.278	0.115	0.673
Alcohol consumption	0.00147	1.001	0.996	1.007	0.00798	1.008	0.995	1.021
Sleep	− 0.2306	0.794	0.609	1.035	0.1506	1.163	0.807	1.674
BMI	− **0.1408**	0.869	0.836	0.903	0.0246	1.025	0.895	1.174

Indicates significant at the 95% level in binary logistics regression

^a^Reference category: non-Black

^b^Reference category: West, Midwest, Northeast

^c^Reference category: Nonmarried

## Discussion

This study focused on within age-related sex heterogeneity in demographic/lifestyle characteristics, health status and healthcare utilization. Disparities in cohort characteristics, prevalence of stroke event and determinants of post-stroke limitation were compared between the two groups. Of the 42,038 women under 60 identified form NHIS data, 1.58% (n = 664) had been diagnosed with stroke compared to 7.44% (n = 1715) of the 23,054 women over 60. While stroke is more common in older women, the National Stroke Association, strokes has reported a rise in stroke incidence among younger adults. Over the past decade, the US has seen a 44% increase in the number of young adults hospitalized due to stroke. Age inclusive studies to date have been primarily mixed gender. This analysis, however, examines within gender differences comparing characteristics, prevalence of stroke and determinants of post-stroke functional limitation between women above and below the age of 60.

Understanding risk factor profiles are critically important to understanding strategies to reduce stroke risk in women. For example, a recent study showed that stroke risk factors are more strongly associated with risk of stroke in women compared to men regardless of the stroke subtype and with men having higher incidence than women [39].

These findings highlight the complexity of the relationship between stroke risk factors and stroke onset particularly among women in general, women of different ages and women of different races. They also suggest more detailed analyses should be completed to examine variability among women as well as differences between men and women. More importantly, comparison of these age cohorts indicates two distinctly different populations of women experiencing stroke. While older women had a higher prevalence of healthcare utilization including blood pressure checks, cholesterol screening, speaking with healthcare providers and taking prescription medications, the young cohort was significantly more likely to visit the emergency or engage in an appointment with a physician. Younger women with stroke were more likely to be Black, reside in the south and be married. However, they were also more likely to smoke and slept significantly fewer hours per night than their older counterparts. These results compare with similar mixed gender and age inclusive assessment of stroke in women and minority populations [40–42]. These findings also highlight previously reported age-related differences in disease prevention practices [43]. Magwood et al. [43] found that although many young stroke survivors exhibit comorbid disease conditions that are similar to older stroke survivors, they are less like to follow-up with physicians than older stroke survivors.

Additional comparisons showed that older women had a significantly higher prevalence of those physical health conditions traditionally related to stroke—diabetes, hypertension and high cholesterol—while the younger cohort reported substantially higher mental health concerns—frequently feeling depressed, worthless or sad. These findings provide a sensible explanation for stroke in the older cohort—increased age coupled with confounding comorbidities and poor health results in stroke among women over 60. Magwood et al. [43] suggest that the higher rate of known risk conditions among young women and women of color could account for these observations. While the patterns of office and ER visits among women under 60 would suggest poor health status, the lower proportion reporting physical health issues could suggest that these issues are present but remain undiagnosed likely due to lack of preventative health services or regular health screening. Interestingly, while this comprises only one plausible explanation, women in poor health have shown to be reluctant to utilize health services for a variety of reasons [44].

A second explanation may be related to a greater presence of vascular risk factors among older stroke women. The role of vascular risk factors has not been widely studied among young women stroke survivors. Cigarette smoking, alcohol consumption and drug use were also associated higher stroke rates particularly among those with confounding chronic or cardiovascular conditions [32, 45, 46]. Age inclusive studies have shown higher rate of stroke among those who have less than high school education and live below poverty level. Numerous studies have shown a correlation between socio economic status (SES) and stroke/stroke mortality—results were robust to the SES indicator used [42, 47]. While not widely studied in adults of any age, being overweight or obese and having inadequate levels physical activity were also more common among individuals with stroke [48, 49].

Young and old women exhibited different predictors of stroke or suspected lifestyle, demographic or environmental characteristic highly correlated with the likelihood of experiencing a stroke According to Carandang et al. [50], risk factors are relevant if they are strong and dose-related, predictive in a variety of samples, pathogenically plausible and supported by other investigations. While relatively few investigations have focused on age specific heterogeneity in women, factors shown to be highly correlated with stroke in this study largely align with those vascular and lifestyle characteristics cited elsewhere [51].

While relevant predictors of stroke were generally consistent in previous research, this cohorts displayed largely different subsets of predictors. For example, risk of stroke increased with the presence of hypertension, diabetes, high cholesterol and high BMI in older women by 19, 23, 53 and 3% respectively. For young women with stroke, both marital status and sleep lower the risk of stroke by 27 and 10% respectively. In contrast, while smoking and alcohol consumption increase the risk for stroke by two and 27%. The older cohort showed increased risk of stroke from primarily health related comorbidities, while the younger cohort shows risk to be more closely related to lifestyle and behavioral characteristics. Overall, demographic/lifestyle predictors of stroke were consistent with previous studies of men and women which have shown that blood pressure, smoking, physical activity and healthy diet reduce the risk of stroke [52, 53]. In fact, longitudinal assessment has demonstrated that modest changes in modifiable lifestyle risk factors can have a substantial effect on risk as all ages.

Black race also emerged as a primary correlate of stroke in younger women and has been previously reported as a risk factor in other studies. Evidence suggests significant racial disparities in stroke and stroke mortality among women with the highest disparities among women aged 50–< 60 [54]. Previous research has shown that hypertension, coronary artery disease, hyperlipidemia and diabetes more common in older Black compared to White adults [55–57]. While the root causes of these disparities is not fully understood, studies suggests a younger age of stroke onset among Black adults [58–60], potentially because of a heavier burden of stroke risk factors among Black adults at younger ages, as early as adolescence [61]. For example, elevated systolic blood pressure (SBP) may confer a greater risk of stroke among Black adults compared with White adults [62]. Importantly, socioeconomic factors have demonstrated only a minimal role in explaining racial disparities in stroke compared with the contribution of traditional stroke risk factors [58, 60]. Although higher socioeconomic status may attenuate racial disparities in stroke, Jiménez, et al. [54] suggests that the elevated stroke incidence among younger Black women and those without a family history of stroke, could reflect persistent racial differences in the distribution of deleterious exposures at the individual, neighborhood and institutional levels across the life course. Unequal access to material and social resources and psychological stress among Black women may result in accelerated cardiovascular aging. While cursory assessment of these disparities may suggest that they could be driven by income or other socioeconomic differences, studies have shown that lower income does not increase risk of ischemic stroke [63]. We do note that previous that showed higher level of income was associated with higher risk of ischemic stroke, the study focused only on individuals over age 65 indicating a reversal of the social gradient. Ultimately, the results could be explained by selective survival, yet no evidence of higher stroke risk among lower socioeconomic groups was found.

A second issue that should be considered in analyses of stroke relates to genetic contribution to stroke risk. To date, studies including genetic disposition have been limited, but family history is suggested to be a risk factor for ischemic stroke [64, 65]. While strokes occurring in very old age tending to be less familial, those occurring at younger ages are believed to be at least partially heritable [66–70]. However, evidence of heritability is not sufficient to assert it comprises the primary cause of stroke in younger adults. However, alternative theories fail to explain the fact that nearly one fourth of strokes occur in people under the age of 65.

Finally, measures of post-stroke function were completed to examine potential differences between older and younger women. Binary indicators for overall functional limitations, stroke problems impeding activity and chronic conditions limiting behaviors used to assess limitations were highly related with physical health determinants in both cohorts. Hypertension, diabetes and high BMI indicate lower levels of general health, reduced functionality and limited activity. All three indicators show a negative relationship to age suggesting that limitations increase over time. Not surprisingly, women taking prescription medication, experiencing surgeries and visiting the emergency show an increased likelihood of limitations and obstructed activity suggesting higher levels of impairment and greater post-stroke limitations.

### Limitations

Although interesting findings, the study does have a number of imitations. First, NHIS did not include respondent age at stroke. Without this information, individuals were classified into age cohorts by their age at the time of the interview rather their age at the event. Therefore, it is possible that older women who experienced a stroke below the age of 60 were misclassified. Second, NHIS does not include an indicator for the totally number of stroke events, the type of stroke suffered, months post-stroke, stroke severity, or the receipt of post-stroke rehabilitative care. These factors were unavailable in the NHIS data but have the potential to confound estimations of functional limitations and other post-stroke outcomes. While data from 5 years of the NHIS data were pooled for this analysis, NHIS is a cross sectional data set precluding longitudinal assessment of individuals over time as they recover from each stroke event. Third, all women in the sample were insured; therefore, results are not generalizable to an uninsured population which could have different outcome correlates. Fourth, all NHIS data included functional status and stroke events are self-reported and, therefore, subject to recall and selectivity bias. Finally, type, quality and the relationship to the primary care has been shown to impact post-stroke outcomes, disability status and quality of life. Unfortunately, caregiver information and type are not included in the NHIS survey. Finally, the NHIS is based on respondent interviews. Survey data is subject to recall, telescoping and social desirability bias.

## Conclusion

Stroke is a major cause of death and disability in the United States impacting 23–29% of the population. While a variety of studies have examined stroke, stroke-related mortality, and post-stroke disability, most current research involves mixed-gender panels. This study exploits the within gender heterogeneity among women with stroke by examining factors related to stroke in young and old women. Results suggest that those factors related to stroke differ between young and old women with behavioral characteristics including smoking frequency, alcohol consumption, and sleep being significant in young women and diabetes, high blood pressure, high cholesterol and high BMI being correlated in older women. However, health status and receipt of healthcare were the most significant determinants of post-stroke functional status for both cohorts. Since those factors correlated with stroke in women vary significantly by age, studies examining women in different age cohorts are needed to address age and gender specific factors and have the best opportunity to reduce or postpone the devastating impact of stroke in women.

## Data Availability

The datasets generated and/or analyzed during the current study are available in the National Health Interview Survey repository, https://www.cdc.gov/nchs/nhis/data-questionnaires-documentation.htm
